# Next Generation Sequencing in Clinical Oncology: Applications, Challenges and Promises: A Review Article

**Published:** 2018-10

**Authors:** Faezeh SHABANI AZIM, Hamidreza HOURI, Zohreh GHALAVAND, Bahram NIKMANESH

**Affiliations:** 1. Student Scientific Research Center, School of Allied Medical Sciences, Tehran University of Medical Sciences, Tehran, Iran; 2. Dept. of Microbiology, School of Medicine, Shahid Beheshti University of Medical Sciences, Tehran, Iran; 3. Zoonosis Research Center, Tehran University of Medical Sciences, Tehran, Iran; 4. Dept. of Medical Laboratory Sciences, School of Allied Medical Sciences, Tehran University of Medical Sciences, Tehran, Iran

**Keywords:** Next generation sequencing, Cancer, Genetic diagnosis, Biomarkers, Personalized medicines

## Abstract

**Background:**

The aim of this mini-review is to highlight the potential applications of next-generation sequencing technology to the field of clinical oncology with respect to genetic diagnosis, cancer classification, predictive biomarkers and personalized medicine.

**Methods:**

Scientific databases were searched to collect relative data.

**Results:**

Effective systematic analysis of whole-genome sequence and whole-exome sequence of tumors, targeted genome profiling, transcriptome sequencing and tumor-normal comparisons can be performed using NGS in order to diagnosis of several types of cancer.

**Conclusion:**

NGS technology can be powerful enough to discover new and infrequent gene alterations, identify hereditary cancer mutation carriers and provide a reliable molecular portrait of wide range of cancers in a quick and cost-effective manner.

## Background

From molecular standpoint, cancer is a complex disease involving a set of genetic alterations and subsequent changes of gene expression patterns that induce uncontrolled proliferation (1). In fact, two cancers with the same histologic origin may be different in the growing, spreading and also treatment response. These differences derived from unique genetic content of each cancer (2). Although DNA is the same in different cells, encoding genes of an organ differ from others. Different types of tumors may have similar DNA, but their gene expression pattern is diverse (3). In this way, understanding the genomic characterization and transcriptomic landscapes of cancer is paving the way of diagnostic, prognostic and predictive biomarkers, leading to shape sustainable and effective treatment for each patient (4). On the other hand, several types of cancer show a familial predisposition and definite gene mutations confer a high-lifetime risk to develop the disease. Therefore, such genetic predisposition can be clarified for several cancer syndromes, for instance, hereditary breast and ovarian cancer, via genetic diagnostic screening methods. NGS not only aids the clinician to select an appropriate therapy which targets the mutated genes but also it can reveal the cause of resistance mechanisms to chemotherapy or targeted therapy.

### Overview of advances in DNA sequencing technologies

The ability to decipher DNA sequences (code of life) is providing scientists with powerful insights into the conceptual foundations of biological and biomedical sciences. Modern sequencing launched in 1977 with the manifestation of first generation sequencing, comprised the chemical cleavage technique which soon fell into disuse and the enzymatic dideoxy chain termination method which became the preferred method due to its high accuracy in targeted sequencing in many large-scale sequencing projects from 1980s until the mid-2000 (5). DNA sequencing by capillary electrophoresis based on Sanger method has opened up the possibility to identify genetic make-up of any biological system (6). This method has been accommodated to obtain long contiguous DNA sequence reads (500 bp to 1 kb in length), which allows for the determination of the size (from 50–1200 bp) of a product through the use of size standards with per-base accuracies of up to 99.999%. Despite having this advantage, applications sequencing approaches in large-scale projects have been restricted inasmuch as it is labor-intensive, time-consuming, and costly (7, 8). The initial effort was made to surpass these obstacles and problems which has led to the birth of second-generation sequencing or next-generation sequencing (NGS) platforms. These innovative technologies have the great potential to sequence many samples in parallel at unprecedented speed and low cost (9). Since the introduction of NGS technology, witnessed tremendous progress have been carried out in genetic research and discovery of human diseases during the last decade. Overall, NGS is an umbrella term refers to any DNA high throughput sequencing technology including Illumina/Solexa, ABI/SOLiD (Sequencing by Oligo Ligation and Detection), 454/Roche, Helicos, etc. (10).

### Application of NGS in genetic analysis of tumors

These new and innovative technologies have opened up the possibility to cost-effectively perform systematic analysis of whole-genome sequence and whole-exome sequence of tumors, targeted genome profiling (sequencing a subset of key genes known associations with cancer), transcriptome sequencing (RNA sequencing of cancer) and tumor-normal comparisons. These applications can help to identify cancer-related variants, including structural rearrangements, copy number alterations (CNAs), point mutations (nucleotide substitutions, small insertions and deletions) and gene expression alterations (11).

### Classification of tumors based upon genetic profiles

The emerging of molecular analysis surmounted the limitations of prevalent solid tumor classification methods according to the morphology of tumor cells and the circumfluent tissue (12). NGS has lately demonstrated the capacity as a cost-effective and high-throughput approach to identify and characterize clinically actionable genetic variants across the large number of genes at unprecedented speed in a single test; such improvements make the use of NGS probable in clinical practice (13). There have been universal efforts underway to catalogue mutations in multiple cancer types through sequencing hundreds of tumors of different subtypes and this is likely to lead to remarkable new discoveries in the search for novel diagnostic, prognostic, and therapeutic targets (14). These new technological advances in molecular profiling, could help physicians attain better accuracy in the classification of human cancers and discovery of it is primary site which are important to obtain better comprehension of cancers and effective therapeutic strategies development (15). Hence, over the last 10 years, our understanding have remarkably increased about the molecular basis of tumor progression and treatment response by sequencing patient genomes and match combination of mutation with specific cancer subtype (4). This method has recently been used successfully employed to the detection and identification of somatic mutations in hematopoietic malignant tumors, solid tumors, and constitutional genetic mutations that cause many of the known hereditary cancer predisposition syndromes in clinical oncology (16). Predictive biomarkers are usually either the direct target of inhibitory drugs (e.g., estrogen receptor & HER2), or molecules involved in DNA repairment (e.g., methylguanine-DNA methyltransferase), or polymorphisms of genes involved in drug metabolism (e.g., thiopurine methyltransferase and uridine glucoronyltransferase). Presently, most successfully targeted therapy in this field are seen in the members of the tyrosine kinase receptor family, epidermal growth factor receptor (EGFR), human epidermal growth factor receptor 2 (HER2) and also non-receptor messenger molecules such as KRAS (17). Currently, the transfer of predictive biomarkers from discovery to clinical practice of personalized oncotherapy have been approved for the management of five diseases: chronic myeloid leukemia, colon, breast, lung cancer and melanoma (Table 1).

**Table 1:** Examples of predictive molecular biomarkers in oncotherapy

***Gene***	***Pathway***	***Cancer types***	***Anticancer Agent***	***Refs***
ERBB2 (HER2)	Receptor tyrosine kinase (ERBB2)	Breast, bladder, gastric & lung cancer	ERBB2 inhibitors	(18, 19)
			ERBB2 antibodies	
MET	RTK (MET)	Bladder, gastric & renal cancer	MET inhibitors	(18, 19)
			MET antibodies	
DDR2	RTK	Lung adenoid cystic carcinoma & lung large cell carcinoma	Some tyrosine kinase inhibitors	(18, 20)
PIK3CA, PIK3R1	PI3K	Breast, colorectal & endometrial cancer	PI3K inhibitors	(18)
PTEN MTOR & TSC1	PI3K mTOR	Numerous cancers Tuberous sclerosis & bladder cancer	PI3K inhibitors	(18, 21)
			mTOR inhibitors	(18, 22)
FGFR1	FGFR1	Myeloma, sarcoma, bladder, bresast, ovarian, lung, endometrial & myeloid cancer	FGFR inhibitors	(18, 19)
			FGFR antibodies	
BRCA1 & BRCA2	(DNA damage repair signaling)	Breast & ovarian cancer	PARP inhibitors	(23, 24)
MRN Complex: (MRE11- RAD50- NBS1)	HR repair pathway (DNA damage repair signaling)	Breast, ovarian, colorectal, gastric, prostate cancer, leukemia & melanoma	MRN complex inhibitors	(25, 26)
ERCC2 (XPD)	NER (Nucleotide Excision Repair Pathway) with ATPase and helicase activity	Breast, ovarian, lung & bladder cancer	Specific DNA repair pathway inhibitors	(18, 26, 27)
KRAS (Also known as V-Ki-ras2 Kirsten rat sarcoma viral oncogene homolog)	RAS/MEK/ERK & PI3K/AKT	Pancreatic, colon, lung, biliary tract, endometrial, cervical, bladder, liver, myeloid leukemia & breast cancer	RAF inhibitors PI3K inhibitors MEK inhibitors	(18, 24, 28)

DDR2 (Discoidin domain-containing receptor 2); PIK3CA (PI3K catalytic subunit-á); PTEN (phosphatase and tensin homolog); mTOR (mammalian target of rapamycin); TSC1 (tuberous sclerosis 1 protein); FGFR1 (Fibroblast growth factor receptor 1); HR (homologous recombination); NS1 (Nijmegen Breakage Syndrome 1); ERCC2 (Excision Repair Cross-Complementing 2); XPD (Xeroderma Pigmentosum complementation D).

### Personalized medicines

With the emerging of next generation technologies, the study of tumor biology has changed more than ever (29). The use of these molecular technological advances showed that from histopathological standpoint, solid tumors have genetic heterogeneity between cancer cells within a tumor (30). In this perspective, the using of traditional “one size fits all” medicine or single drug-regimen for patients with signs and symptoms of the same cancer is undesirable and could lead to drug resistance, unnecessary toxicities and costs (31). Increasing our understanding of molecular cell biology of cancers resulting in the development of several molecularly targeted therapies. Tailor-made therapy in cancer also called personalized cancer treatment means optimizing medicines to individuals based on molecular properties of tumor and tissues surrounding its environment as well as patient’s characteristics. In this way, using molecular diagnostics is far critical to characterize patient’s genetic make-up and understanding how his tumor growth (17). Despite the great trend toward precision medicines, currently a few molecular biomarker-based targeted therapies with proven efficiency are available for routine use in the clinic settings. Some examples of such targeted approach include erlotinib as EGFR-inhibitor for EGFR-mutant lung tumors, and vemurafenib for BRAF-mutant melanoma (32).

## Conclusion

Several early attempts were carried out to accommodate these high-throughput genetic technologies in clinical cancer settings, such impressive advances of sequencing technologies in recent years allow oncologists and physicians to access comprehensive and cost-effective understanding of molecular aspects of tumor growth and metastasis to help them investigate the etiology and pathogenesis of cancer and identify novel therapeutic targets. This improves the quality of diagnostic services in clinical lab and subsequently enables health care to deliver specific treatment match to a patient’s cancer which promises to have fewer toxic side effects over existing medicines as well as astonishing outcomes.

## Ethical considerations

Ethical issues (Including plagiarism, informed consent, misconduct, data fabrication and/or falsification, double publication and/or submission, redundancy, etc.) have been completely observed by the authors.
